# Procyanidin B2 induces apoptosis and autophagy in gastric cancer cells by inhibiting Akt/mTOR signaling pathway

**DOI:** 10.1186/s12906-021-03225-1

**Published:** 2021-02-24

**Authors:** Yuqin Li, Xiaolan Lu, Peiying Tian, Kai Wang, Jianping Shi

**Affiliations:** Department of Gastroenterology, Shanghai Pudong Hospital, Fudan University Pudong Medical Center, No.2800 Gongwei Road, Pudong New District, Shanghai, 201399 China

**Keywords:** Gastric cancer, Procyanidin B2, Apoptosis, Autophagy, Akt, mTOR

## Abstract

**Background:**

Procyanidin B2 (PB2), a unique component of the grape seed and other medicinal plants. PB2 has shown wide anticancer activity in various human cancer cells. However, it remains unclear about the biological effects and associated mechanisms of PB2 on gastric cancer cells.

**Methods:**

Cell proliferation was measured by CCK8 assay, and cellular lactate dehydrogenase (LDH) release was measured in the culture medium. Cellular apoptosis was observed via TUNEL staining assay and measured by caspase-3 and -9 activities. Autophagy was observed by LC3 staining. Western blot analysis was performed to verify autophagy-associated proteins (Beclin1 and Atg5) and Akt-mTOR pathway.

**Results:**

PB2 reduced the viability of BGC-823 and SGC-7901 cells in a concentration-dependent manner. Furthermore, PB2 induced increased apoptosis rate of gastric cancer cells and enhanced caspase-3 and -9 activities. Simultaneously, PB2 triggered autophagy in gastric cancer cells, with enhanced LC3 staining and increased expression of Beclin1 and Atg5, while the inhibition of autophagy by 3-MA reversed the PB2-induced suppression on cell viability. In addition, PB2 significantly decreased p-Akt and p-mTOR protein expression of gastric cancer cells.

**Conclusion:**

PB2 exerts anti-proliferative and apoptotic effects and induces autophagy by modulating Akt/mTOR signaling pathway. PB2 may be developed as a potential therapeutic drug for gastric cancer.

## Background

Gastric cancer is a malignant disease that ranks the fifth most common malignancies in the world [1]. It was estimated 679,100 new cases diagnosed with gastric cancer and 498,000 deaths occurred in 2015 [2]. Resection only benefits certain patients, but shows transient effect on advanced-stage gastric cancer, which often demonstrates metastasis. Currently, the main treatment for advanced gastric cancer is chemotherapy, but the treatment outcome remains unsatisfactory [3]. This is caused by the fact that gastric cancer cells show resistance to most chemotherapeutic drugs [4]. Therefore, novel effective anticancer drugs are urgently needed to identify for cancer therapy.

Apoptosis and autophagy are two important biological process that are associated with cell growth, survival and metastasis [5]. Apoptosis is a type of programmed cell death with characterized morphology, such as cell shrinkage and DNA fragmentation [6]. Autophagy is a catabolic process that maintain intracellular homeostasis through degrading cellular components like misfolded proteins and damaged organelles [7]. Apoptosis and autophagy can be induced in the prevention of tumor growth of gastric cancer cells [8], and the related mechanisms involve PI3K/Akt/mTOR pathway [9, 10]. However, there are complex interactions between apoptosis and autophagy in tumors, including gastric cancer cells. Autophagy could play a pro-death role or pro-survival role in the gastric cancer cells, which is dependent on different upstream signals [11, 12]. Moreover, Akt/mTOR mediates the therapeutic effects of many natural herbs’ extracts on gastric cancer. For example, Pectolinarigenin induced gastric cancer cell apoptosis and autophagy through inhibiting PI3K/Akt/mTOR pathway [13]. Sophocarpine activated cell apoptosis, induced autophagy, and down-regulation of PI3K/AKT cell survival pathway in gastric cancer cells [14]. However, the recurrence and mortality rates of gastric cancer are still high [15]. Hence, it is a promising therapeutic strategy to targeting the signaling pathways of PI3K/Akt/mTOR for cancer treatment.

Procyanidins are flavonoids extracted from many plants like grape seed, apples and cocoa beans [16], with the B-type procyanidins (PB2) as the most common form of procyanidins [17]. It has reported that there is negative correlation between the consumption of procyanidins and the risk of cardiovascular diseases, T2DM and cancers [18]. PB2 demonstrates anti-cancer activity in Hodgkin’s lymphoma [19], breast [20] and prostate cancer [21]. However, the anti-cancer activity and related molecular mechanism of PB2 on gastric cancer remains unclear.

In this study, we explored the effects of PB2 on growth inhibition, apoptosis and autophagy of gastric cancer cells using CCK-8 assay, lactate dehydrogenase (LDH) release assay, TUNEL staining detection and western blot analysis. To explore the detailed mechanisms of PB2, the role of autophagy in the effects of PB2 in gastric cancer cells was investigated.

## Methods

### Cell culture

Human gastric cancer cell line BGC-823 and SGC-7901 were purchased from the Cell Bank of Chinese Academy of Sciences (Shanghai, China). Both cells were cultured in RPMI-1640 medium with 10% fetal bovine serum (Gibco, Grand Island, NY, USA) at 37 °C and 5% CO_2_. After cells grew to 80–90% confluence, they were passaged. The medium was replenished every 2 days. The Procyanidin B2 were purchased from MedChemExpress (MCE, Cat. No: HY-N0796) and dissolved in Dimethyl sulfoxide (DMSO;1 mM) and stored at − 20 °C.

### Cell viability assay

BGC-823 and SGC-7901 cells (1 × 10^4^ cells/well) were seeded into 96-well plates, and were incubated with varying concentrations of PB2 (0, 10, 20, 50, 100 and 200 μM) for 48 h, or incubated with PB2 (50 μM) and autophagy inhibitor 3-MA for 48 h. Cells were added with 10 μL of CCK-8 solution (Sigma-Aldrich; Merck KGaA) in each well for 3 h incubation, and the absorbance was examined at 450 nm by a microplate reader.

### LDH release assay

Cell injury in BGC-823 and SGC-7901 cells was evaluated by measuring the amount of LDH that released into the culture medium from damaged cells. After treatments with various concentrations of PB2 for 48 h, the medium was collected by centrifugation at 3000×g for 10 min, and the amount of LDH was detected by spectrophotometry via an LDH assay kit (Beyotime, Cat. No: C0016). Cellular LDH content was expressed as U/dL.

### TUNEL assay for apoptosis

SGC-7901 cells were fixed with 4% paraformaldehyde for 30 min, washed twice with phosphate buffer saline (PBS), blocked with 3% H_2_O_2_ for 10 min, and were permeabilized with 0.1% Triton X-100 for 2 min on ice. Then cells were stained with 50 μL TUNEL solution and incubated at 37 °C in the dark. Cells were then stained with DAPI solution for 30 min to mark nucleus. After washing 3 times in PBS, the cells were mounted in mounting medium and were observed under a fluorescence microscope (515–565 nm; Olympus, BX60, Japan) (magnification, × 200). At least 1000 cells that selected from five random visual fields were counted to determine the number of TUNEL+ cells.

### Caspase-3 and caspase-9 activity assay

After various treatments, SGC-7901 cells were harvested and lysed (100 mL lysis buffer for 15 min on ice), and supernatant was obtained. Then, 10 μL supernatants were mixed with 90 μL AC-DEVD-pNA (Caspase-3, Beyotime, Cat. No: C1115) or AC-LEHD-pNA (Caspase-9, Beyotime, Cat. No: C1158) for 2 h at 37 °C to measure caspase-3 and caspase-9 activities, respectively. Absorbance at 405 nm was measured by a microplate reader. The caspase-3 and caspase-9 activities in each group was normalized to those values of control group.

### Autophagy

Following treatment with PB2 for 48 h, SGC-7901 cells were washed with PBS, fixed with 4% paraformaldehyde (pH 7.4), blocked with 1% BSA, and permeabilized with 0.1% Triton X-100. Cells were incubated with goat polyclonal anti-LC3-II antibody (Cat. No. sc-398,822; Santa Cruz, CA, USA) at 4 °C overnight, followed by incubation with FITC-linked secondary antibody for 1 h at 37 °C. Then cells were stained with DAPI (10 mg/mL), and observed under a fluorescence microscope (IX71, Olympus, Japan). The number of LC3 positive cells (punctate fluorescence) was counted, and its percentage to total cells (DAPI fluorescence) was calculated.

### Western blot

Total protein was extracted from SGC-7901 cells. The protein samples (50 μg) were loaded in 10% SDS-PAGE and then transferred to a PVDF membrane. Subsequently, the membrane was incubated with rabbit antibodies against Beclin1 (ab62557, 1:2000 dilution; Abcam, Cambridge, UK), ATG5 (ab108327, 1:2000 dilution; Abcam, Cambridge, UK), pan-AKT (ab18785, 1:1000 dilution; Abcam, Cambridge, UK), phospho S473 AKT1 (ab81283 1:1000 dilution; Abcam, Cambridge, UK); mTOR (ab2732; 1:1000 dilution; Abcam, Cambridge, UK), phospho S2448 mTOR (ab84400; 1:1000 dilution; Abcam, Cambridge, UK), and β-actin (ab8227, 1:2000 dilution; Abcam). After washing with TBS, the membrane was incubated with HPR-linked secondary antibody (1:250). The bands were observed by a chemiluminescent detection system (Thermo Scientific, Waltham, MA, USA). The optical density of the protein bands was normalized to β-actin, and analyzed using ImageJ software.

### Statistical analysis

Data were expressed with means ± standard deviation (SD), and analyzed by SPSS 20.0 statistical software (SPSS Inc., Chicago, IL, USA). The differences between three or more groups were analyzed by One-way ANOVA, with post hoc testing for pairwise comparison. *P* < 0.05 was considered as statistical significance.

## Results

### PB2 inhibited human gastric cancer cell growth and induced damage

**Fig. 1:**
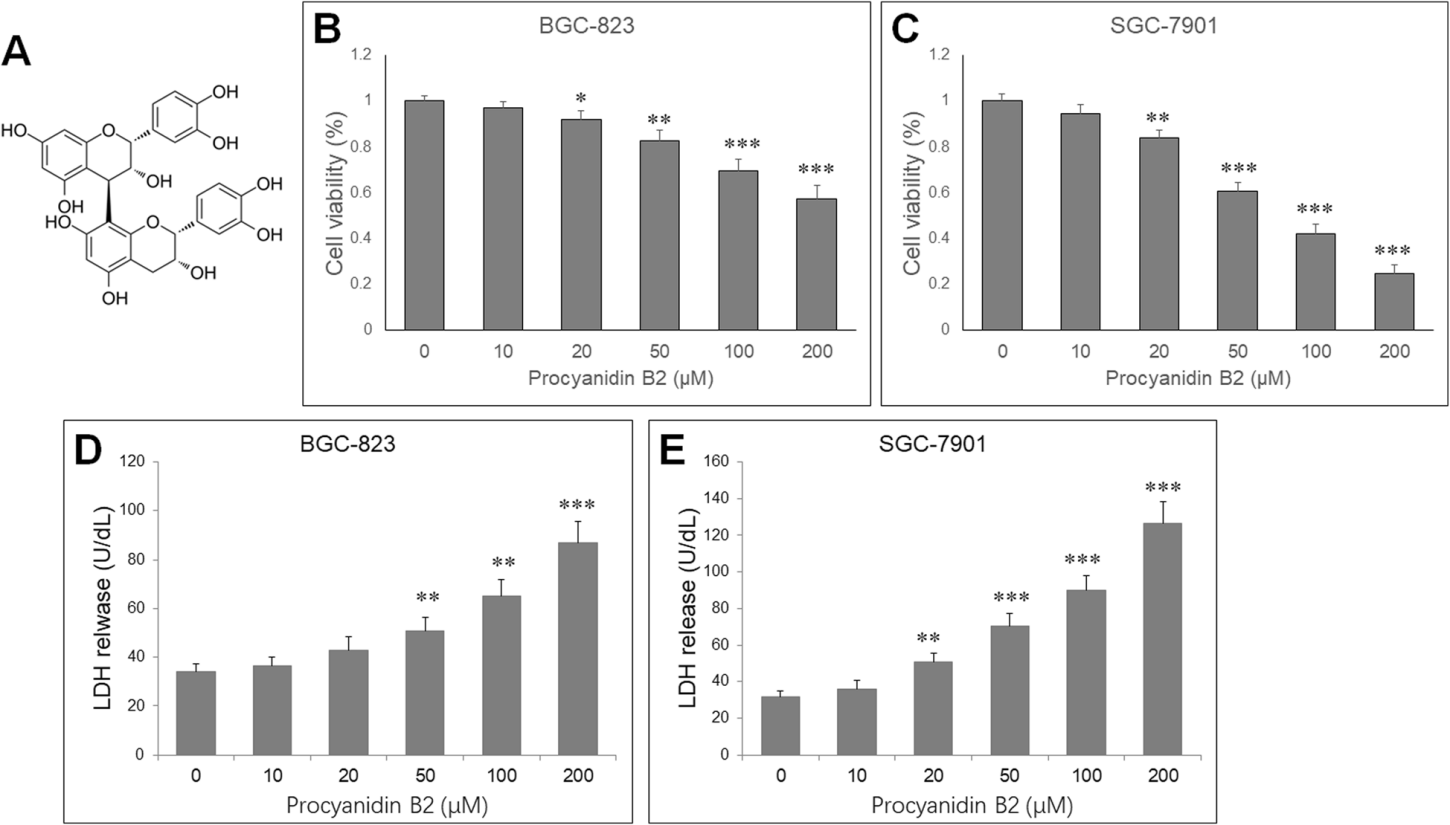
PB2 reduces cell viability of gastric cancer cells. **a** Chemical structure of PB2. BGC-823 **b** and SGC-7901 **c** cells were treated with PB2 (0, 10, 20, 50, 100 and 200 μM) for 48 h. Cell viability was measured by a CCK-8 assay. **d** BGC-823 and **e** SGC-7901 cells were incubated with PB2 for 48 h, and the culture media were collected to measure LDH release by colorimetry. Data are represented as the mean ± standard deviation. *P < 0.05, **P < 0.01, ***P < 0.001 vs. control group. PB2, Procyanidin B2; LDH, lactate dehydrogenase

To evaluate the growth inhibitory effect of PB2 (Fig. 1a), the cell viability was determined using the CCK-8 assay. BGC-823 and SGC-7901 gastric cancer cell line were treated with PB2 (0, 10, 20, 50, 100 and 200 μM) for 48 h. PB2 significantly reduced cell viability of these two cells (Fig. 1b, c). The LDH in culture media were measured and showed that PB2 treatment for 48 h induced LDH release from BGC-823 and SGC-7901 cells compared with the untreated cells (Fig. 1d, e). Based on these preliminary results, SGC-7901 is more sensitive to PB2 and was selected in following experiments, and 0, 20, 50 and 100 μM were selected as the appropriate concentrations.

### PB2 induced the apoptosis of SGC-7901 cells

**Fig. 2:**
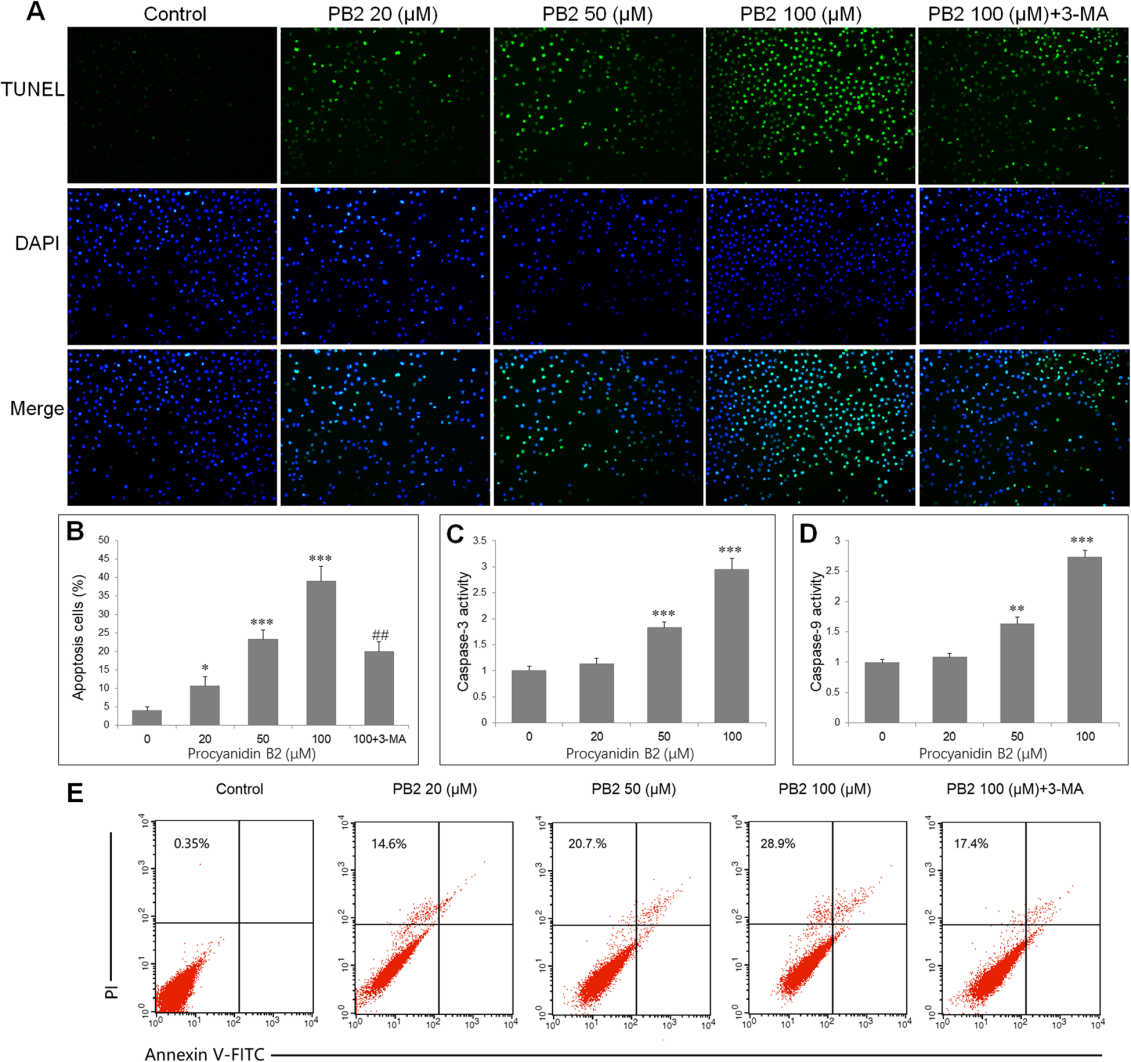
PB2 induces apoptosis of gastric cancer cells. SGC-7901 cells were incubated with PB2 (0, 20, 50 and 100 μM) or PB2 with 3-MA (100 μM) for 48 h. **a** SGC7901 cells were stained with TUNEL (green) and DAPI (blue). Representative photomicrographs are shown (scale bar: 5 μm). **b** Quantification of TUNEL+ cells. The caspase-3 **c** and caspase-9 **d** activities were determined. **e** Apoptotic rate was determined by flow cytometry using double staining of Annexin-V FITC and PI. The number in the left upper quadrant indicates the apoptotic rate, which is the sum of early apoptosis (right lower quadrant) and late apoptosis (right upper quadrant). ***P < 0.001 vs. control group; ##*P* < 0.01 vs. PB2 100 μM group

SGC-7901 cells were treated with PB2 (0, 20, 50 and 100 μM) or PB2 with 3-MA (100 μM) for 48 h, and TUNEL and DAPI staining assays were applied to assess apoptotic cell death. The control cells manifested weak homogeneous blue fluorescence in nucleus. The PB2-treated cells exhibited shrinkage of cell and nucleus and brighter green fluorescence (TUNEL stain), which are morphological characteristics of apoptotic cells (Fig. 2a). Quantification of TUNEL staining results showed that PB2 increased apoptosis rate (TUNEL positive) of SGC-7901 cells, and the apoptosis rate can be reduced by coincubation with 3-MA (Fig. 2b). Then caspase-3 and caspase-9 were analyzed by colorimetric method. PB2 treatment increased caspase-3 and caspase-9 activities in SGC-7901 cells (Fig. 2c, d). Moreover, the apoptotic rate was measured by Annexin V-FITC/PI staining. PB2 increased apoptotic rate of SGC-7901 cells in a concentration dependent manner, which can be attenuated by cotreatment with a autophagy inhibitor 3-MA (Fig. 2e). The results indicate that PB2 promotes gastric cancer cells apoptosis.

### PB2 induced autophagy in SGC-7901 cells

**Fig. 3:**
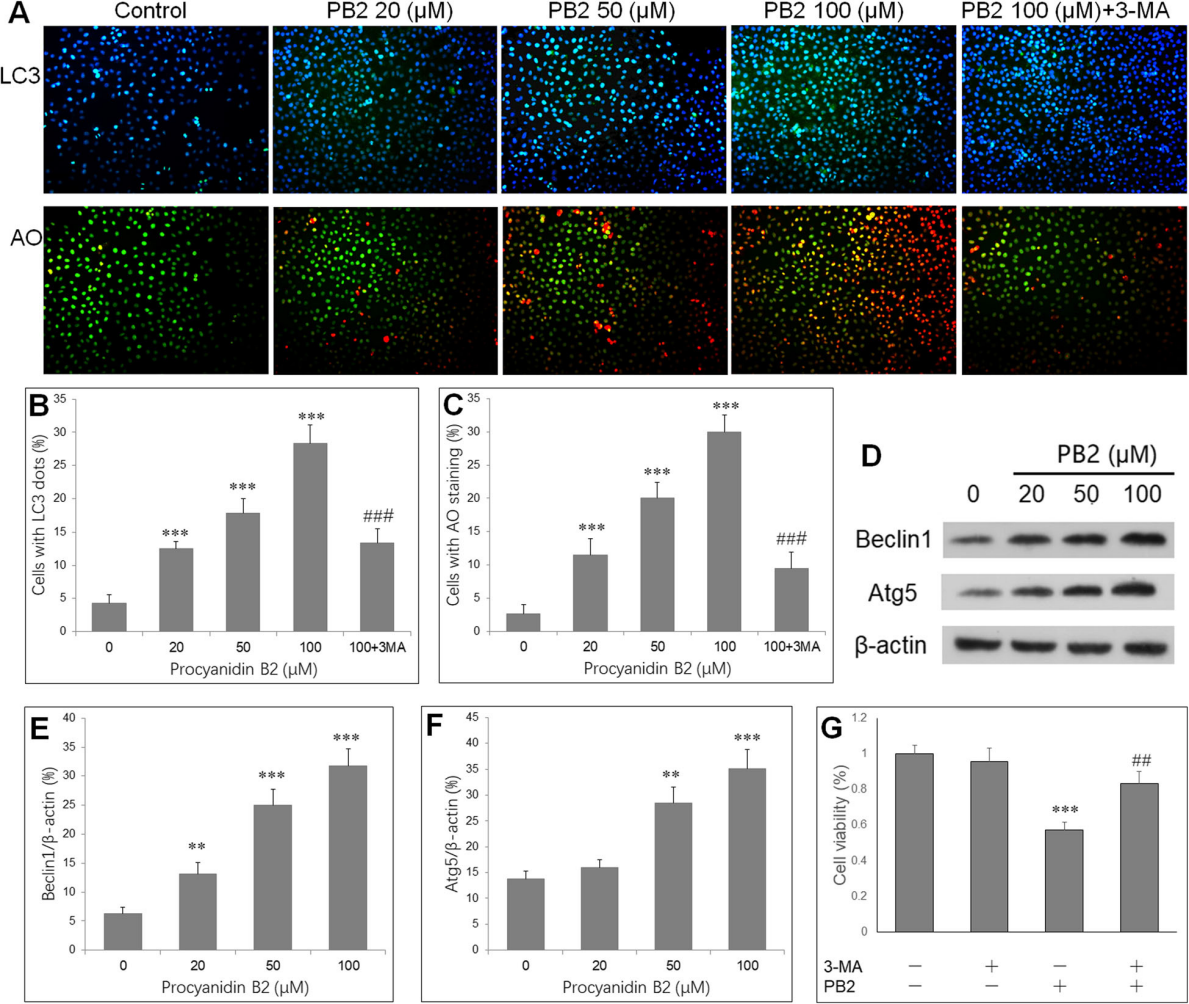
PB2 induces autophagy in gastric cancer cells. Cells were incubated with different concentrations of PB2 for 48 h. **a** Cells were stained with LC3 antibody or acridine orange (AO), and observed under a fluorescence microscopy (green, × 200). **b** Quantification of cells with LC3 dots. **c** Quantification of cells with AO staining. **d** Western blot shows the protein expression of autophagy-associated proteins, Beclin1 and Atg5. PB2 increases the mean relative levels of Beclin1 **e** and Atg5 **f** in SGC-7901 cells. Calculation of all these proteins were normalized to β-actin. **g** PB2-induced reduction in cell viability is reversed by 3-methyladenine (3-MA). SGC-7901 cells were treated as follow: Control, 3-MA (100 μM), PB2 (50 μM), 3-MA + PB2 for 48 h. Then, cell viability was measured by CCK-8 assay. *P < 0.05, **P < 0.01, ***P < 0.001 vs. control group. ##*P* < 0.01, vs. PB2 group

SGC-7901 cells were treated with PB2 (0, 20, 50 and 100 μM) or PB2 with 3-MA (100 μM) for 48 h. SGC-7901 cells were stained with LC3 antibody and DAPI, or stained with acridine orange (AO) and Hoechst 33258, and then observed under a fluorescence microscopy. The DAPI-positive blue puncta represent nucleus, and the merged yellow images are indicators of autophagosomes. Similarly, the nucleus showed green (stained with Hoechst 33258) and the AO-positive red images are indicators of autophagosomes (Fig. 3a). PB2 induced autophagy in SGC-7901 cells, as evidenced by increased yellow fluorescence in cells with PB2, especially in high concentration. Quantification analysis showed that PB2 significantly increased the percentage of autophagic cells (cells with LC3 dots: Fig. 3b; cells stained with AO, Fig. 3c). However, coincubation with 3-MA and PB2 significantly reduced percentage of autophagic cells compared to cells with PB2 alone. This indicated that PB2 dose-dependently promotes autophagy in SGC-7901 cells which can be suppressed by 3-MA. Then autophagy-related proteins Beclin1 and Atg5 were detected by Western blot, and their protein expression were increased by PB2 (Fig. 3d, e, f). Then 3-MA, an autophagy inhibitor, was applied to SGC-7901 cells to explore the role of autophagy in effects by PB2. The CCK-8 results showed that 3-MA notably reversed the inhibitory effect of PB2 on SGC-7901 cells. However, 3-MA alone did not show significant influence on cell viability (Fig. 3g). This indicates that autophagy might play a pro-apoptosis role in PB2-treated gastric cancer cells.

### PB2 inhibited Akt/mTOR pathway in SGC-7901 cells

**Fig. 4:**
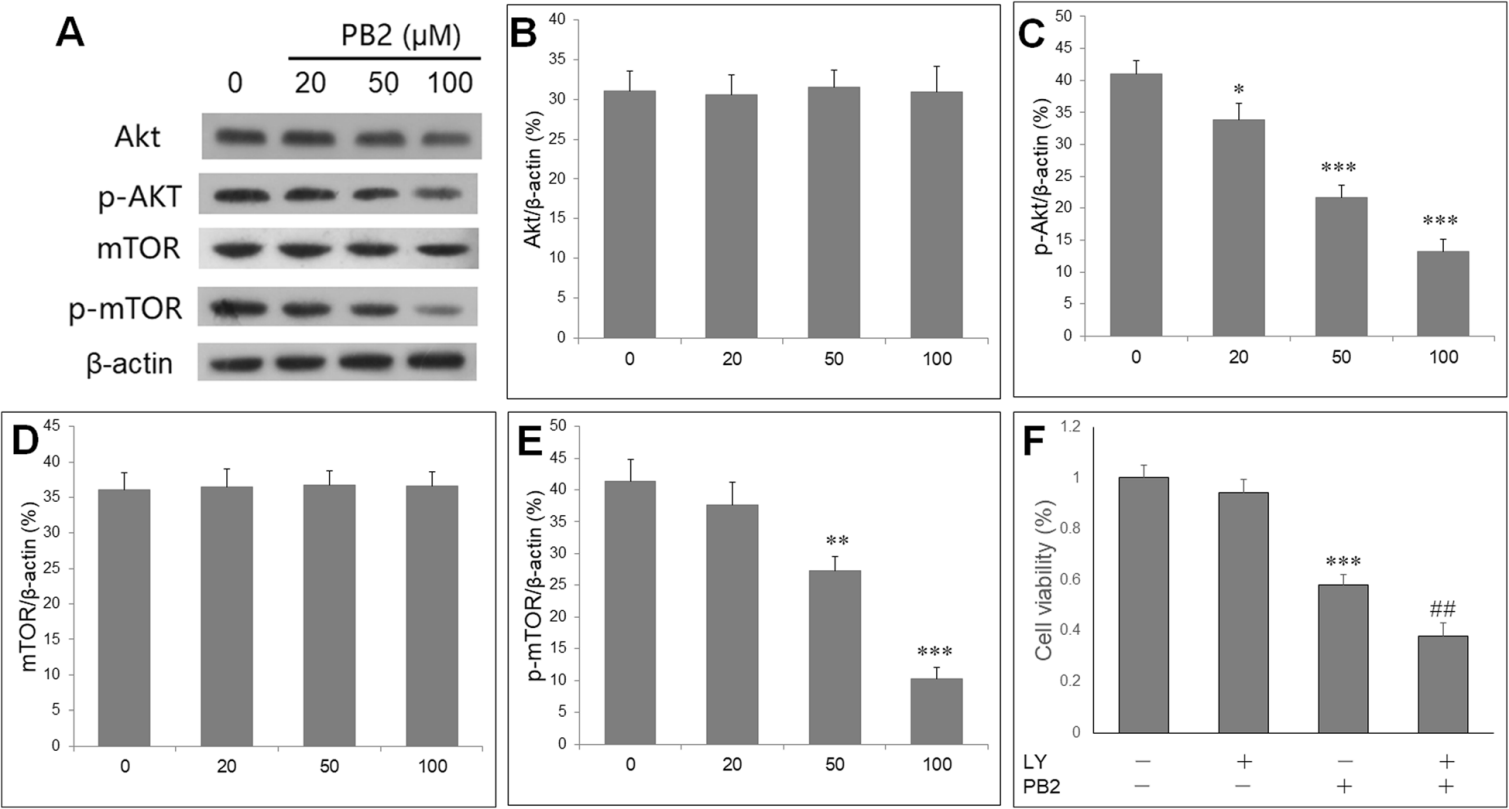
PB2 inhibits Akt/mTOR pathway in gastric cancer cells. SGC-7901 cells were incubated with PB2 (0, 20, 50 and 100 μM) for 48 h. **a** Western blot was carried out to determine Akt, p-Akt, mTOR and p-mTOR proteins. **b** PB2 did not change the expressions of Akt, **c** but reduced p-Akt protein expression in a concentration-dependent manner. **d** mTOR remain unchanged after PB2 treatment, **e** but p-mTOR protein expression is reduced by PB2 in a concentration-dependent manner. ***P < 0.001 vs. ox-LDL group. **f** PB2-induced reduction in cell viability is further enhanced by Akt inhibitor LY294002. SGC-7901 cells were divided into following groups: Control, LY294002, PB2, LY294002 + PB2, and were cultured for 48 h. Cell viability was detected by CCK-8 assay. ***P < 0.001 vs. control group; ###*P* < 0.001 vs. PB2 group

We then explore whether PB2 regulate Akt/mTOR pathway, which involve the process of autophagy. Western blot was performed to determine the protein expression of Akt, p-Akt (Ser473), mTOR and p-mTOR (S2448) (Fig. 4a). PB2 did not change the total Akt but prominently repressed phosphorylated Akt expressions (Fig. 4b, c). Also, PB2 did not change the total mTOR protein but reduced the phosphorylated mTOR expressions (Fig. 4d, e). In order to explore the role of Akt/mTOR pathway in PB2- in cell viability reduction induced by PB2 in gastric cancer cell, Akt inhibitor LY294002 was applied. PB2 (50 μM) significantly inhibited the cell viability of SGC-7901 cells, and this effect was further aggravated by LY294002. Meanwhile, LY294002 alone had no effect on the viability of SGC-7901 cells (Fig. 4f). The results indicated that suppression of Akt/mTOR pathway may mediate reduced cell viability by PB2 in gastric cancer cells.

## Discussion

In this study, we showed that PB2 induces apoptosis and autophagy in GC, and to our knowledge, this is the first research to identify anti-tumor effect of PB2 on gastric cancer. Furthermore, PB2 caused autophagic cell death via inhibition of Akt/mTOR signaling pathway. Autophagy inhibition by specific inhibitor 3-MA showed increased cell viability in PB2-treated gastric cancer cells. These results indicate that PB2 regulates apoptosis and autophagy via PI3K/Akt/mTOR pathway.

PB2 has shown certain pharmacological and biochemical effects in various cancer cells, but its anticancer effect has received limited investigation. PB2 has been reported to inhibit cell viability in Hodgkin’s lymphoma [19], breast cancer [20] and prostate cancer cells [21]. These results indicated that PB2 increased apoptosis and altered the signaling pathway such as NF-κB. However, the effect of PB2 on gastric cancer cells is unclear. In this study, we tested the anti-tumor effects of PB2 on BGC-823 and SGC-7901 cells, which are poorly differentiated and moderately differentiated gastric cancer cells, respectively. This study firstly reports that PB2 markedly inhibited cell proliferation of BGC-823 and SGC-7901 cells, with reduced cell viability and increased LDH release in culture media. PB2 showed no cytotoxic effect on normal keratinocytes (HaCat) up to 200 μM concentration [22], which is high above the used concentrations in our experiment. This indicates low toxicity of PB2 as a potent antitumor agent in gastric cancer. Furthermore, grape seed proanthocyanidin, including PB2, prevented colon carcinogenesis by suppression on cell proliferation and survival [23]. A derivative of PB2 could target cancer stem cells (CSCs) in prostate cancer through inhibition of sphere formation and self-renewal of CSCs [24]. Therefore, PB2 might serve as an effective means for preventing gastric cancer carcinogenesis.

Our study showed increased LDH content in culture media of gastric cancer cells after PB2 treatment. LDH is an enzyme that promotes glycolytic process by converting pyruvate to lactate. A common metabolic change of cancer cell is elevated glycolysis, so LDH is highly expressed in multiple cancers and is associated with malignant progression [25]. Gastric cancer patients also showed high serum LDH level, which was associated with shorter survival time [26]. Moreover, cell apoptosis can cause the destruction of cell membrane structure, and then lead to the release of intracellular enzymes into the culture medium. As a relatively stable enzyme, LDH is released from the cell into the culture medium, which can form a visual color reaction. Therefore, the cytotoxicity of PB2 can be quantitatively analyzed by measuring the LDH activity in the culture medium [27]. A recent study showed that PB2 gallate suppressed glycolytic enzyme of activated T cells by reducing LDH expression at the posttranscriptional level [28]. Whether PB2 modulate the LDH expression in gastric cancer cells remains unclear, and deserves further investigation.

Our study showed that the growth inhibitory effect of PB2 might be due to apoptotic cell death. Caspases are a group of cysteine-containing proteolytic enzymes and classified either as initiators (caspases − 2, − 8, − 9 and − 10) or executors (caspases-3) of apoptosis in cancer cells. The initiator caspases enter the apoptotic cascade in the early stage and then activates the executor caspases [29]. In the present study, it was shown that the activities of executor caspase-3 and initiator caspase-9 were increased following PB2 treatment. Similar results of PB2 on tumor cell apoptosis were also observed in human colon cancer cells [30].

This study showed the increased autophagy of SGC-7901 cells after PB2 treatment. Moreover, the protein levels of Beclin1 and Atg5, two reliable marker of autophagy [31, 32], were significantly increased by PB2. In addition, autophagy is closely related to the development and therapy of tumor, which is dependent on different upstream signals [11, 12]. Our results showed that an autophagy inhibitor 3-MA and PB2 co-treatment increased cell viability and attenuated apoptosis compared to cells treated with PB2 alone, and suggests that autophagy plays a pro-death role in PB2-treated gastric cancer cells. Although autophagy is a major type of cell death other than apoptosis, autophagy plays dual roles by promoting cell death and survival in many disease processes. Moreover, there are complex crosstalk between autophagy and apoptosis, which is regulated by various intermediate molecules and pathway, such as mTOR, Beclin-1 and Atg5 [33]. Autophagy also plays both pro-survival and pro-death roles in gastric cancer. Helicobacter pylori infection can reduce autophagy of gastric mucosa, which involves carcinogenesis by Helicobacter pylori [34]. Our results are consistent with other reports that the apoptosis induced by procyanidins or PB2 in human hepatoma and colorectal cancer cells were both reversed by 3-MA [35, 36]. As an important risk fact of gastric cancer, Helicobacter pylori infection in gastric mucosa leads to a decrease in autophagy, and subsequently promotes initiation of gastric cancer [37]. This indicates that PB2 might serve as a promising chemopreventive agent in gastric cancer through enhancing autophagy. However, additional data are required to confirm this hypothesis.

Our results showed that Akt/mTOR signaling is also a regulated pathway by PB2 in gastric cancer, as evidenced by decreased expression of p-Akt and p-mTOR in PB2-indeuced SGC-7901 cells. PI3K-Akt-mTOR signaling pathway is frequently activated in gastric cancer patients, and can promote the release of free Bcl-2 from Bcl-2/Bcl-xl dimer, and promote the proliferation and growth of tumor [38]. PI3K-Akt-mTOR is an important autophagy-related signal pathway, and regulates the crosstalk between autophagy and apoptosis in various tumors [39]. This study showed that PB2 decreases the expressions of p-Akt and p-mTOR in gastric cancer cells, and similar results were also observed in colorectal cancer cells from other report [36]. Therefore, PB2 might promote the apoptosis and autophagy of gastric cancer cells though could inhibition of PI3K-Akt-mTOR signaling pathway.

There are some limitations of our study. Firstly, we provided only in vitro evidence to support the anti-cancer effects of PB2 on gastric cancer cells. However, it remain further study to investigate in vivo effects in animal models. PB2 could inhibit the activation of hepatic stellate cells in vitro and in vivo, with a half-maximal inhibitory concentration (IC50) as 82.56 μM [40]. Our in vitro study showed that the IC50 of PB2 was > 200 μM in BGC-823 and 50–100 μM in SGC-7901 cells. So it seems that PB2 might also showed in vivo effects on some gastric cancer cells, such as xenografted SGC-7901 cells in mice. Secondly, though simultaneous induction of apoptosis and autophagy were observed in gastric cancer cells by PB2, their complex regulatory interaction and upstream regulatory signaling pathways of Akt-mTOR remains unclear.

## Conclusion

PB2 inhibits viability and promotes apoptosis and autophagy of gastric cancer cells through Akt/mTOR signaling pathway. This study provides PB2 as a new potential agent for the prevention and therapy of gastric cancer.

## Abbreviations

CSCs: Cancer stem cells
CCK-8: Cell counting kit-8
LDH: Lactate dehydrogenase
PB2: Procyanidin B2
